# Effects of single dose of dexamethasone on patients with systemic inflammatory response

**DOI:** 10.1590/S1516-31802006000200008

**Published:** 2006-03-02

**Authors:** Domingos Dias Cicarelli, Fábio Ely Martins Benseño, Joaquim Edson Vieira

**Keywords:** Sepsis syndrome, Sepsis, Inflammation, Adrenal cortex hormones, Dexamethasone, Síndrome séptica, Sepse, Inflamação, Corticosteróides, Dexametasona

## Abstract

**CONTEXT AND OBJECTIVE:**

Systemic inflammatory response syndrome (SIRS) is a very common condition among critically ill patients. SIRS, sepsis, septic shock and multiple organ dysfunction syndrome (MODS) can lead to death. Our aim was to investigate the efficacy of a single dose of dexamethasone for blocking the progression of systemic inflammatory response syndrome.

**DESIGN AND SETTING:**

Prospective, randomized, double-blind, single-center study in a postoperative intensive care unit (Surgical Support Unit) at Hospital das Clínicas, Faculdade de Medicina, Universidade de São Paulo.

**METHODS:**

The study involved 29 patients with SIRS. All eligible patients were prospectively randomized to receive either a single dose of 0.2 mg/kg of dexamethasone or placebo, after SIRS was diagnosed. The patients were monitored over a seven-day period using Sequential Organ Failure Assessment score (SOFA).

**RESULTS:**

The respiratory system showed an improvement on the first day after dexamethasone was administered, demonstrated by the improved PaO_2_/FiO_2_ ratio (p < 0.05). The cardiovascular system of patients requiring vasopressor therapy also improved over the first two days, with a better evolution in the dexamethasone group (p < 0.05). Non-surviving patients presented higher lactate assays than did survivors (p < 0.05) during this period.

**CONCLUSIONS:**

Dexamethasone enhanced the effects of vasopressor drugs and evaluation of the respiratory system showed improvements (better PaO_2_/FiO_2_ ratio), one day after its administration. Despite these improvements, the single dose of dexamethasone did not block the evolution of SIRS.

## RESUMO

**CONTEXTO E OBJETIVO:**

A síndrome da resposta inflamatória sistêmica (SRIS) acomete muitos pacientes internados em unidades de terapia intensiva. A evolução destes pacientes com SRIS para sepse, choque séptico e síndrome da disfunção de múltiplos órgãos (SDMO) pode conduzi-los rapidamente para o óbito. A proposta do trabalho é avaliar a eficácia da dexametasona em dose única como tratamento da SRIS.

**TIPO DE ESTUDO E LOCAL:**

Estudo prospectivo, aleatório, duplamente encoberto, realizado na Unidade de Terapia Intensiva pós-operatória (Unidade de Apoio Cirúrgico) do Hospital das Clínicas da Faculdade de Medicina da Universidade de São Paulo.

**MÉTODOS:**

Foram estudados 29 pacientes com diagnóstico de SRIS. Os participantes foram aleatoriamente divididos em dois grupos que receberam dexametasona (0,2 mg/kg em dose única) ou placebo após o diagnóstico de SRIS. Os pacientes foram acompanhados durante sete dias de internação na UTI através do escore SOFA *(Sequential Organ Failure Assessment)*.

**RESULTADOS:**

Os pacientes que receberam dexametasona apresentaram melhora do sistema respiratório no primeiro dia, com aumento da relação PaO_2_/FiO_2_ (p < 0,05). Entre os pacientes que faziam uso de vasopressores, os que receberam dexametasona tiveram diminuição da necessidade destas medicações nos primeiros dois dias após a dose de dexametasona (p < 0,05).

**CONCLUSÃO:**

A dexametasona diminuiu a necessidade de medicações vasopressoras e causou aumento da relação PaO_2_/FiO_2_ no primeiro dia após sua administração. Apesar destes efeitos, a dexametasona em dose única não bloqueou a evolução dos pacientes com SRIS.

## INTRODUCTION

Systemic inflammatory response syndrome (SIRS) is a very common condition among critically ill patients. It occurs frequently in the postoperative period, with or without infection. SIRS may be related to trauma, burns, pancreatitis or pulmonary diseases, leading to acute lung injury (ALI) and acute distress respiratory syndrome (ARDS).^1^ SIRS can be defined by two or more symptoms such as fever (body temperature > 38°C) or hypothermia (< 36°C), tachycardia (> 90 beats/min), tachypnea (> 20 breaths/min) or hyperventilation (PaCO_2_ < 32 torr), and abnormal white blood cell counts (> 12,000 cells/µl or < 4,000 cells/µl) or immature neutrophils (bands > 10%).^1,2^

SIRS, sepsis, septic shock and multiple organ dysfunction syndrome (MODS) are strongly related. The patient's progression through this sequence often leads to death. However, some patients with SIRS develop MODS without diagnosed infection or sepsis.^3^ Sepsis is defined as a condition in which the patient displays the SIRS criteria as well as a documented or a suspected infection. Severe sepsis is defined as sepsis with organ dysfunction, inadequate perfusion or hypotension (systolic blood pressure < 90 mmHg or a reduction ≥ 40 mmHg from the baseline). Septic shock is defined as severe sepsis with hypotension despite adequate fluid resuscitation, which requires vasopressor support. MODS is defined as organ dysfunction in critically ill patients who require intervention to reach homeostasis.^1^

Activation of the inflammatory cascade by a new agent, with or without infection, seems to be self-sustained.^3^ However, resolution of the inducing agent cannot be the only treatment for SIRS and cannot break the progression of the inflammatory response that leads to MODS and death. Despite early administration of antibiotics, the progression of SIRS to sepsis, septic shock, MODS and death is sometimes unavoidable.

To lessen the progression of SIRS and improve the outcome, drugs such as glucocorticoids and anti-inflammatory nonsteroids have been used, albeit unsuccessfully. More recently, specific monoclonal antibodies against inflammatory cytokines such as tumor necrosis factor (TNF) have been tested.^4^

Glucocorticoids have an important immunosuppressive effect, in which they reduce the transcription of pro-inflammatory genes by inhibiting the nuclear factor kappa B.^4-8^ Several studies have involved the use of corticosteroids to reduce the systemic inflammatory process associated with the host response to sepsis and septic shock.^9-21^ However, most of these studies involved extremely high doses over short periods (< 24 hours), and no diagnostic criteria for sepsis were applied, because such criteria were not yet well-established at that time. The results were controversial, although some authors believed in the benefit of corticosteroids after observing early shock reversal or blood pressure elevation in treated patients.^11,13,14,17,19,20,22,23^

Some of these studies have not been confirmed by other groups.^15^ The results from two meta-analyses indicated no survival benefit when supraphysiological doses of corticosteroids were administered for short-term treatment of sepsis, and higher infection rates were associated with corticosteroids.^12,18^ Some authors^16,24,25^ believe that more careful, broad-er-scope studies are needed to conclusively identify the real benefits from these drugs.

Several reports have been published recently, from studies involving lower doses of hydrocortisone. These showed improved outcomes for patients with septic shock, and also showed that methylprednisolone could be used to obtain ARDS resolution.^10,21,26,27^ These recent results, as well as the unfavorable results from using specific monoclonal antibodies, rekindle hope for the efficacy of corticosteroids in treating SIRS.

## OBJECTIVE

This study aimed to evaluate the effectiveness of a single dose of dexamethasone in blocking the progression of SIRS.

## METHODS

This study was prospective, randomized, double-blind and placebo-controlled. After approval by a local ethics committee, informed consent was obtained from patients or from their next of kin prior to enrollment.^28^ Twenty-nine patients admitted into the postoperative intensive care unit (Surgical Support Unit, SSU) of Hospital das Clínicas, Universidade de São Paulo, took part in the study. Apart from these patients, one other patient was excluded after his next of kin withdrew their consent.

Patients with SIRS diagnosed 12 hours after SSU admission,^1,29^ with or without sepsis, were eligible for the study. Patients were excluded if they were under 18; had a history of immunosuppression therapy or a history of glucocorticoid use for over two weeks within the last year or upon admission to this hospital; were suffering from active pancreatitis; had a terminal illness (end-stage neoplasm with a life expectancy of less than three months); or had recently suffered gastrointestinal hemor- rhage.^27^ After SIRS diagnosis, blood, urinary and catheter-tip cultures (if infection was suspected) were obtained in accordance with the SSU hospital routine. A randomization table determined the order of inclusion for the patients to receive placebo, among the expected 30 admissions. All the eligible patients were prospectively randomized into two groups: Group D comprising 15 patients and Group P with 14 patients. Group D patients were given intravenous dexamethasone 0.2 mg/kg (in a single dose),^30^ while Group P patients received placebo (0.9% physiological saline solution).

Baseline severity of illness was assessed by means of the Acute Physiology and Chronic Health Evaluation II score (APACHE II).^31,32^ After SIRS diagnosis, the patients were assessed daily for seven consecutive days using the Sequential Organ Failure Assessment score (SOFA),^33-37^ or until their discharge from the SSU. Lactate and C-reactive protein plasma concentrations were also measured daily.^38^

The patients received conventional therapy regarding antibiotic regimens, serial blood cultures (whenever their body temperature was > 38° C) and discharge criteria. Appropriate clinical and laboratory tests were conducted daily throughout the study. The subjects were evaluated during their stay in the SSU on the basis of the duration of vasopressor support (SOFA score of two or more for the cardiovascular system), mechanical ventilation and mortality.

Statistical analysis was performed using the Sigma Stat for Windows software, 2.03 version (SPSS Inc.). For continuous variables, the treatments were compared using the Student t test, Mann-Whitney U test and two-way ANOVA (analysis of variance) for treatment and outcome conditions.

## RESULTS

The mean age (± standard deviation, SD) of the 29 patients was 53 ± 19 years (range: 18 to 77 years). The study involved 19 males and 10 females (66% versus 34%). The mean age (± SD) for Group D was 51 ± 22 years, while the mean age for Group P was 54 ± 14 years. There was no difference between these groups in relation to APACHE II (15 ± 5 for Group D and 16 ± 4 for Group P). At the baseline, the demographic characteristics and severity of disease were similar in the two groups (Table 1).

**Table 1:** Baseline characteristics of the 29 patients with diagnosed systemic inflammatory response syndrome

Characteristics	Placebo Group (n = 14)	Dexamethasone Group (n = 15)
Age (years)	54 ± 14	51 ± 22
Male sex (%)	64.3	66.7
Weight (kg)	67.2	69.3
APACHE II score	16 ± 4	15 ± 5
SOFA score	6.9	7.1
Prior or preexisting conditions (%)		
Hypertension	28.6	33.3
Myocardial infarction	14.3	13.3
Diabetes	14.3	13.3
Liver disease	7.1	–
COPD	7.1	6.7
Cancer	21.4	20
Recent trauma	35.7	20
Mechanical ventilation	64.3	60
Shock (use of any vasopressor)	50	60

*APACHE = Acute Physiology and Chronic Health Evaluation; SOFA = sequential organ failure assessment; COPD = chronic obstructive pulmonary disease.*

No statistical difference was found in either the mortality rates for the groups during the seven-day follow-up period (five deaths in Group D and three deaths in Group P; p = 0.682; Fisher exact test), or in the blood, urinary or catheter-tip cultures. With regard to collateral effects from dexamethasone (increased glucose, secondary infections or gastrointestinal hemorrhage), only one patient in Group P developed gastrointestinal hemorrhage (patient 7, with enterectomy due to intestinal perforation) while two patients in Group P developed pneumonia (patient 6, with colectomy due to neoplasia, and patient 7, with aneurysm repair).

Among the 29 patients with SIRS, 14 failed to reach the SIRS criteria on the second day of their stay at the SSU. Eleven patients showed positive blood cultures, suggesting that these 38% of the patients had sepsis. Nine patients (31%) had septic shock and the remaining 10 patients required vasopressor therapy during their SSU stay.

**Figure 1:**
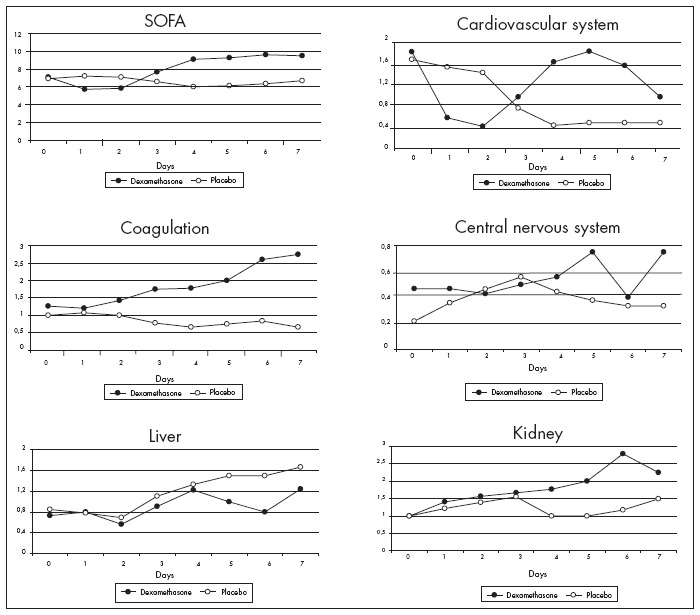
Progression of organ dysfunction in 29 patients with systemic inflammatory response syndrome, as assessed using the different components of the Sequential Organ Failure Assessment score (SOFA).

The two groups showed similar SOFA scores during the study. No differences were found in coagulation disorders (platelet counts), hepatic dysfunction (serum bilirubin), renal dysfunction (serum creatinine), or central nervous system dysfunction according to the Glasgow scale (Figure 1).

**Figure 2:**
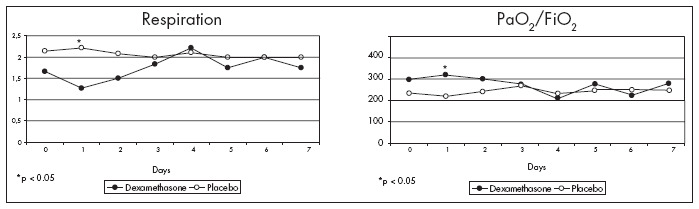
Improvement in the respiratory system and better evolution of the PaO_2_/FiO_2_ ratio in the first day after diagnosis of systemic inflammatory response syndrome in patients that received a single dose of dexamethasone, compared with those who received placebo.

The respiratory system 24 hours after dexamethasone administration showed an improved PaO_2_ /FiO_2_ ratio (Mann-Whitney test; p = 0.017). However, this improvement did not persist throughout the study (Figure 2). The duration of mechanical ventilation was the same in the two groups (3.26 ± 2.46 days for Group D and 3.64 ± 3.15 days for Group P).

**Figure 3:**
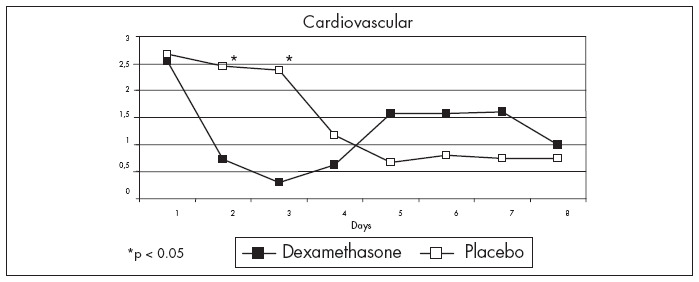
Improvement in the cardiovascular system during the first and second days after diagnosis of systemic inflammatory response syndrome in the group of patients that received a single dose of dexamethasone, compared with placebo, including only the patients that received vasopressor therapy.

The cardiovascular system score showed a trend towards improvement in Group D over the first two days (Figure 1). The better evolution in Group D, when the patients who did not receive vasopressor therapy were excluded (Mann-Whitney test; p = 0.007 and p = 0.018 on days 1 and 2, respectively) (Figure 3), was noteworthy. However the duration of vasopressor therapy was statistically similar for the two groups (2.2 ± 2.1 days for Group D and 2.8 ± 1.9 days for Group P).

All the 29 patients were also divided into two additional groups: survivors and non-survivors, in relation to the treatment. Eight patients (27.6%) died during the seven-day period (SSU mortality). After using two-way ANOVA (analysis of variance) for the treatment, the cardiovascular system score was high for 48 hours among the non-survivors of Group P. In fact, these measurements displayed a significant difference (p = 0.028 on day one; p = 0.003 on day two). The respiratory system score showed the same pattern, i.e. it was low for 48 hours among survivors of Group D, with a significant difference (p = 0.0038 on day one; p = 0.008 on day two).

**Figure 4:**
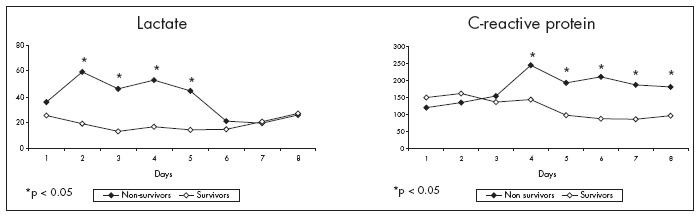
Evolution of lactate (mg/dl) and C-reactive protein (µg/ml) in the survivor and non-survivor groups of patients with systemic response syndrome.

Compared with the survivor group (21 patients), the non-survivors presented higher lactate assays (Mann-Whitney test; p = 0.002) for four days during the study (Figure 4).

C-reactive protein was higher in the nonsurvivor group, starting on day three (p = 0.028) and remaining high throughout (Figure 4). There was no difference between Groups D and P relating to C-reactive protein.

Among the 29 patients studied, 17 patients (58.6%) had suspected infection (nine patients in Group P and eight patients in Group D), and positive blood cultures were found in 11 (37.9%) (six patients in Group P and five patients in Group D). Of the 29 patients, 12 (41.3%) were given prophylactic antibiotics and five had to receive therapeutic antibiotics; 17 (58.6%) received therapeutic antibiotics and three had to change antibiotics.

## DISCUSSION

Despite recent studies in which patients with septic shock were treated with hydrocortisone, the present study has revealed some advantages in the use of dexamethasone^39^. This drug was chosen because of its potency and long-lasting action (36-48 hours), and its higher anti-inflammatory and lower mineralocorticoid effects. In comparison with hydrocortisone, dexamethasone causes no changes in sodium reabsorption and does not interfere in the water balance, thus avoiding hypervolemia and sodium disturbances.^30^ No recent study was found involving the use of dexamethasone in SIRS or septic patients. All things considered, it seemed reasonable to test dexamethasone on the basis of a single dose, to investigate its benefits and observe any possible adverse effects.

The pathophysiology of sepsis includes host inflammatory response, endothelial damage, increased coagulation with decreasing fibrinolysis, fibroproliferation and microclot formation and relative adrenal insufficiency.^40^ However, this systemic inflammatory response can lead to organ dysfunction, instead of protecting and regulating homeostasis.^40^

Corticosteroids can improve the effects of vasopressor drugs, thus reestablishing receptor sensitivity, with better effects from the use of lower doses.^23,26^ The first explanation for the hemodynamic improvement of patients receiving corticosteroids was based on observations of the relative adrenal insufficiency that they might develop.^23,25,41^ In addition, some published reports have shown that patients without relative adrenal insufficiency could display better evolution following corticosteroid therapy.^42,43^ These reports may serve as backing for our finding of early discontinuation of vasopressor therapy in patients receiving dexamethasone.

Currently, the recommendations for corticosteroids in relation to sepsis are that this class of drugs should be used during refractory septic shock, but not during severe sepsis in the absence of shock or with mild shock.^44^ Whether or not sepsis is the systemic inflammatory response to infection, sepsis, severe sepsis and septic shock constitute different gradations in the continuum of a disease process. As this process continues, it is correlated with increasing organ dysfunction and mortality. Therefore, early infusion of corticosteroids to block this process that began with an inflammatory reaction ought to be tested.

The use of corticosteroids in septic patients can be explained by the relative adrenal insufficiency of these patients. However, it seems to us that the principal mechanism of action of corticosteroids is based on their anti-inflammatory effect.

An experimental study revealed that corticosteroids decreased pulmonary edema and collagen formation.^45^ Another study demonstrated an improvement in patients with ARDS after corticosteroid therapy, probably because of the inhibition of pulmonary fibroproliferation.^27,46^ These previous studies support our observation that patients treated with dexamethasone displayed a better PaO_2_/FiO_2_ ratio on the first day after therapy. However, the use of corticosteroids for treating the early phase of ALI/ARDS has not been recommended (the recommendations include only the fibroproliferation phase).^47^ Nonetheless, even the patients in the present study who received dexamethasone during the early exudative phase (days 1-5) of ALI/ARDS showed an improved PaO_2_ /FiO_2_ ratio. The rationale for this may include the observation that the integrity of the epithelial barrier in relation to the resolution of alveolar edema appears to be a determining factor in the outcome for ARDS patients. Patients who can concentrate the protein in the edematous fluid during the first 12 hours of illness are more likely to recover than those who cannot. Finally, since the change in the PaO_2_/FiO_2_ ratio following initial treatment of ARDS could pre-discriminate between survivors and non-survivors,^47^ the use of corticosteroids in the early phases of ALI/ARDS might be considered a reasonable step.

The arterial lactate assays for the survivor group went on decreasing from the first day of the study onwards. This result confirms previous findings that established that lactate is a good marker for septic patients.^48^

On the other hand, C-reactive protein did not appear to be a good marker for the patients' progression, since the non-survivor group showed higher values only after day 3 of the study. Evaluations of infected patients showed no increased levels of C-reactive protein, contrary to what was suggested by other authors.^38,49-52^ Our data cannot support the suggestion that C-reactive protein is a marker for infection. No correlation was observed between C-reactive protein values and the severity of infection or organ dysfunction level.^53^

This study remains part of an ongoing line of research, because of the significant results observed during the first two days after the single dexamethasone dose. Therefore, intravenous dexamethasone will be repeated at 48-hour intervals, to confirm the benefits for patients over a longer period, under closer assessment of their health status.

## CONCLUSIONS

Sepsis and acute lung injury can trigger an increased inflammatory response that appears to be attenuated by the administration of dexamethasone. SIRS treatment with corticosteroids may be not a simple resurrection of last rites,^43^ but a change in therapy that may have been used incorrectly in the past and may now get a fresh start based on new pathophysiological concepts regarding sepsis.

A single dose of dexamethasone enhanced the effects of vasopressor drugs for an apparently temporary period, and an evaluation of the respiratory system also revealed improvements, but it did not block the evolution of SIRS.
